# A Rare Case of Streptococcus pasteurianus Endocarditis Signaling Colon Cancer

**DOI:** 10.7759/cureus.48328

**Published:** 2023-11-05

**Authors:** Gabriela R Gospodinova, Liliya Demirevska, Ivaylo Daskalov, Gabriel D Dimitrov

**Affiliations:** 1 Cardiology, Military Medical Academy, Sofia, BGR; 2 Neurology, Military Medical Academy, Sofia, BGR

**Keywords:** streptococcus bovis, endocarditis, colon cancer, streptococcus gallolyticus, streptococcus pasteurianus

## Abstract

Infective endocarditis (IE) is a life-threatening condition often associated with various complications. A unique subset of IE cases involves the *Streptococcus gallolyticus* group, which has a well-documented but sometimes poorly understood association with colonic neoplasms. Specifically, colon cancer has a well-established association with IE caused by *S. gallolyticus* subspecies (spp.) *gallolyticus*. However, finding colon cancer in cases of IE due to *S. gallolyticus *spp. *pasteurianus* is rather unexpected. Herein, we present a rare instance of IE caused by *S. gallolyticus* spp. *pasteurianus* in a 62-year-old male, which led to the discovery of an underlying colorectal carcinoma. Considering the overall elevated risk of colon cancer in patients with endocarditis and in patients with *S. bovis*/*S. equinus* complex (SBSEC) bacteremia, we decided to proceed to colonoscopy, which revealed adenocarcinoma. The patient was administered a targeted antibiotic regimen and underwent a successful surgical resection, followed by valve replacement surgery. The outcome of this case supports the recommendation of routine colonoscopic evaluation in patients diagnosed with *S. gallolyticus* bacteremia, including those with subspecies pasteurianus, particularly when there are other associated findings. It strengthens the argument for conducting a colonoscopy in individuals diagnosed with SBSEC endocarditis, while carefully considering the specifics of each clinical situation. Our report highlights the need for heightened clinical vigilance and an integrated approach to treatment in similar cases.

## Introduction

The *Streptococcus bovis*/*Streptococcus equinus* complex (SBSEC) is an entity of species residing in the intestinal flora of healthy humans and animals [1]. Although these species are commensal, it is a long-known fact that they can be associated with severe diseases. The first reported identification of *S. bovis* endocarditis in human dates back over 75 years [2]. Subsequently, McCoy and Mason first suggested an association between *S. bovis* endocarditis and colon carcinoma in 1951 [3]. Since then, numerous studies have not only corroborated this association but have also identified connections between SBSEC infection and other conditions.

Traditionally, SBSEC species have been categorized into three biotypes based on their biochemical characteristics: biotype I, biotype II.1, and biotype II.2. With the advent of DNA methods, a new classification system has emerged two decades ago. *S. bovis *biotype I was reclassified as *S. gallolyticus* subspecies (spp.) *gallolyticus* (SGG), biotype II.1 as *S. gallolyticus* spp. *infantarius* (SGI) and *S. gallolyticus* spp. *lutetiensis* (SGL), and biotype II.2 as *S. gallolyticus* spp. *pasteurianus* (SGP) [4].

A refined classification aids in clinical distinction since some subspecies are more commonly associated with specific conditions. Notably, SGG (biotype I) is now known to be more strongly associated with colon cancer and endocarditis than the other subspecies [5]. By contrast, the incidence of colon cancer in biotype II infections appears to be indistinguishable from that in the general population [6-9]. Therefore, the occurrence of both colon cancer and infective endocarditis (IE) is less expected and rare with SGP (biotype II.2).

Herein, we present the case of a 62-year-old male with SGP-associated endocarditis, which led to the discovery of an occult colon cancer.

## Case presentation

A 62-year-old male was admitted to our hospital with a one-week history of fatigue, generalized malaise, and fever. His medical background included arterial hypertension, hyperlipidemia, and atrial fibrillation, for which he was regularly medicated. The patient was a long-term smoker. He denied engaging in any other substance use. In addition, he reported no recent history of travel. On examination, his temperature was 38.5 °C, and he had a Levine III/VI pansystolic murmur best audible at the apex. Vital signs were stable, with a blood pressure of 115/74 mmHg, a heart rate of 85 beats per minute, a respiratory rate of 18 breaths per minute, and an oxygen saturation of 94% on ambient air. The neurological examination was unremarkable.

**Figure 1:**
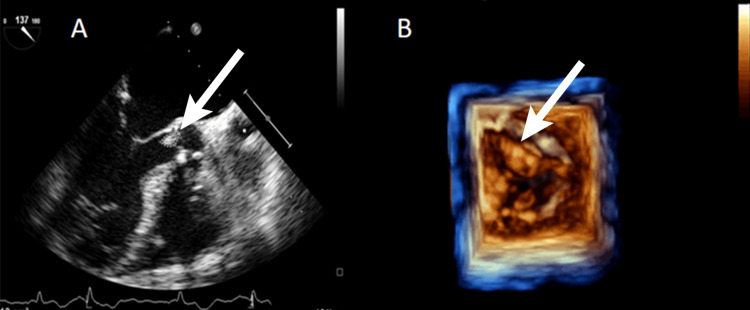
Echocardiogram showing vegetation over the aortic valve. (A) Transesophageal echocardiogram. (B) 3D echocardiogram.

Initial laboratory findings included a hemoglobin level of 116 g/L, a white blood cell count of 15,900/μL with 79% granulocytes, a platelet count of 286,000/μL, and C-reactive protein (CRP) level of 125 mg/L. Both renal and liver functions were within the normal limits. A transthoracic echocardiography revealed vegetations on the aortic and mitral valves, significant aortic valve regurgitation, and moderate mitral valve regurgitation. These findings were further confirmed by a transesophageal echocardiography, which showed mobile tip vegetations over both the mitral and aortic valves (Figure 1).

The patient was started on empirical broad-spectrum antibiotic therapy. Separate blood cultures later identified SGP. Two major clinical Duke criteria were met, therefore confirming definite IE.

A total of four sets of blood cultures, each comprising an aerobic and an anaerobic bottle, were meticulously obtained at standard intervals of approximately 30 minutes, recommended for optimal detection of bacteremia. Two sets were collected prior to the commencement of antimicrobial therapy, and subsequent sets were obtained in line with the standards of good clinical practice. Each set received 9-10 ml of blood, allocated evenly for aerobic and anaerobic cultivation. The cultures were processed using the BD BACTEC FX 40 (Becton Dickinson Diagnostic System, USA), following the manufacturer’s guidelines. The bottles with positive results underwent Gram staining and were subsequently subcultured using standard methods. The isolated pathogen was identified with the VITEK® 2 Compact (bioMerieux, France), and verification was achieved with MALDI-TOF mass spectrometry (MS VITEC, bioMerieux). The antimicrobial susceptibility was assessed through the VITEK® 2 Compact (bioMerieux), with the results interpreted based on the criteria set by the European Committee on Antimicrobial Susceptibility Testing (EUCAST V 8.0) (Table 1). The initial blood cultures were indicative of biotype II.2 (SGP), and an antibiotic regimen was tailored to intravenous vancomycin at a dosing of 15 mg/kg every 12 hours and meropenem at 1 g every eight hours for a planned duration of 14 days. The patient’s fever subsided five days after initiating the antibiotic regimen, and a marked decrease in inflammatory markers was observed with a CRP level of 44 mg/l. Repeat blood cultures obtained on day 7 post-initiation were negative, indicating microbiological resolution of the infection.

**Table 1:** Antimicrobial susceptibility profile Results of the antimicrobial susceptibility testing for the isolated pathogen. The susceptibility is denoted as "R" for resistant and "S" for susceptible.

Antimicrobial	Susceptibility
Amikacin	R
Amoxicillin	R
Amoxicillin/Clavulanic acid	R
Ampicillin	R
Ampicillin/Sulbactam	R
Azithromycin	S
Ceftriaxon	R
Ciprofloxacin	R
Clarithromycin	S
Clindamycin	S
Doxycyclin	R
Erythromycin	R
Gentamicin	R
Levofloxacin	R
Moxifloxacin	S
Pefloxacin	R
Penicillin G	R
Teicoplanin	S
Tetracyclin	R
Tobramycin	R
Trimethoprim/Sulfamethoxazole	R
Vancomycin	S
Meropenem	S

Considering the established association between SBSEC infection and colon cancer, a colonoscopy was performed on the last day of the planned antibiotic therapy. The procedure identified a cecal mass measuring 2.0 x 2.0 cm, a sigmoid mass of 2.0 x 3.0 cm, and two sessile polyps in the descending colon.

**Figure 2:**
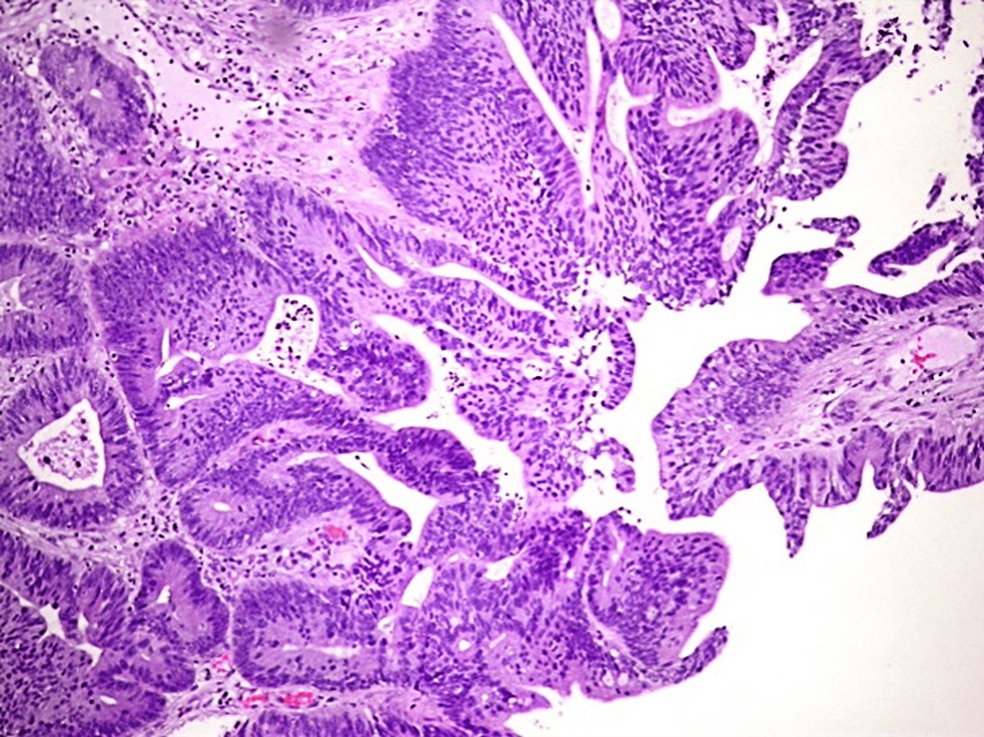
Pathological examination of the resected tumor indicating a moderately differentiated adenocarcinoma with multiple hyperplastic polyps in the descending colon.

The histopathological examination revealed an adenocarcinoma (Figure 2). The cecal tumor was staged as T2N0M0 and the sigmoid tumor as T3N0M0, indicating localized disease without regional lymph node involvement or distant metastasis.

After a multidisciplinary team discussion comprising a cardiologist, a gastrointestinal surgeon, an oncologist, and a cardiothoracic surgeon, it was decided to proceed with surgery to remove the tumors. A meticulous surgical field preparation was followed by a total median laparotomy. The inspection of the abdominal cavity revealed the following: 1) a tumor formation in the mid-third of the sigmoid colon measuring 2.0 x 3.0 cm, preoperatively verified as adenocarcinoma; a second tumor formation in the cecum measuring 2.0 x 2.0 cm suspected of malignancy; several polyps of varying sizes along the colon; 2) the liver was free of any pathologic lesions; 3) no macroscopic pathological changes were observed in the remaining organs.

A sequential mobilization of the distal segment of the ileum, ascending colon, right flexure, transverse colon, left flexure, and descending colon was performed with selective ligation of the ileocolic artery and vein, right colic, middle colic, left colic, and inferior mesenteric vessels. The sigmoid colon was transected in its distal third. The terminal ileum was transected, and continuity was restored with an ileo-sigmoid anastomosis on two levels, using continuous polydioxanone (PDS) 4-0 and 5-0 sutures. The surgical field was lavaged and meticulously secured for hemostasis. Three abdominal drains were placed, directed respectively to 1) the left (upper to the left lateral canal) and lower (toward the small pelvis and anastomosis) and 2) right (in the right lateral canal). The abdominal wall was closed in layers with continuous sutures. The skin was closed, and a dry sterile dressing was applied.

The intervention was performed at the end of the third week of hospitalization. The same antibiotic regimen was continued preoperatively and postoperatively to minimize the risk of surgical site infections, as recommended by the latest guidelines for antibiotic prophylaxis in colorectal surgery. The total antibiotic exposure for the patient was 28 days.

The patient's follow-up recommendations included clinical and laboratory evaluations on postoperative days 7 and 14, with additional follow-up scheduled at the third month and a colonoscopy within the next three years to assess recovery and monitor for any signs of recurrence.

Following the colectomy and the initial evaluations, which were normal, the patient underwent aortic and mitral valve replacement at a third-party center. The patient did not return on the third month and was lost to follow-up.

## Discussion

IE presents with a constellation of symptoms and findings, included in the Duke criteria, recently updated and known as the 2023 Duke-International Society for Cardiovascular Infectious Disease (ISCVID) [10-12]. While IE is the foremost consideration when a patient presents with fever, fatigue, and a new-onset heart murmur, physicians must consider a range of differential diagnoses when evaluating patients with similar clinical presentations. Various valvular disorders, such as mitral valve prolapse or aortic stenosis, can mimic endocarditis symptoms, including heart murmurs. Echocardiography is crucial to differentiate between these conditions. Myocarditis can present with fever, chest pain, and heart dysfunction, mimicking endocarditis symptoms; however, the underlying pathology is different. Certain malignancies, such as lymphomas or leukemia, can infiltrate the heart and cause valvular dysfunction, leading to symptoms similar to endocarditis. Infections at other sites in the body, such as the skin, bones, or urinary tract, can lead to systemic symptoms and fever, which may mimic endocarditis. Conditions, such as systemic lupus erythematosus (SLE) and vasculitis, can present with fever, fatigue, and multiorgan involvement, potentially mimicking endocarditis. This uncertainty again highlights the importance of using established algorithms, such as the Duke-ISCVID criteria, rather than a purely subjective assessment. The diagnosis of IE in our patient was established based on clinical manifestations, blood cultures, laboratory findings, and cardiac imaging, fulfilling the Duke-ISCVID criteria for a definite diagnosis.

A population-based cohort study found that patients with endocarditis had an increased risk of developing colorectal and other cancers. The risk was higher in the first five years after endocarditis diagnosis [13]. Acknowledging this fact and considering the long-known association between SBSEC infection and colon cancer [14], we performed colonoscopy, which revealed cancerous lesions. A timely and accurate diagnosis is crucial for managing potentially life-threatening conditions and their underlying causes. In this particular case, a multidisciplinary approach was essential to address both conditions.

We did a thorough search in PubMed, Web of Science, and ScienceDirect, using the keywords (“biotype II.2” OR “biotype II/2” OR “pasteurianus”) + “endocarditis,” and found only four case reports of SGP endocarditis, associated with colon cancer [15-18].

This scarcity could be related to the fact that colon cancer incidence in patients infected with biotype II subspecies, including SGP (biotype II.2), appears similar to that in the general population in multiple studies [6-9]. By contrast, SGG (biotype I) is strongly associated with colonic malignancies (Table 2) [5]. SGP and SGI branches on the other side are predominantly associated with immunosuppressive conditions, polymicrobial bacteremia, and hepatobiliary and urinary tract diseases. SGP has also been implicated in meningitis, particularly in infants [19].

**Table 2:** The four principal species of Streptococcus bovis/Streptococcus equinus complex (SBSEC; previously known as group D streptococci) and their association with colorectal cancer. The association of *Streptococcus gallolyticus* spp. *gallolyticus* (biotype I) with colorectal cancer is stronger than that of *Streptococcus gallolyticus* spp. infantarius, *Streptococcus gallolyticus* spp. *lutetiensis* (biotype II.1), and *Streptococcus gallolyticus* spp. *pasteurianus* (biotype II.2) [5].

Phenotypic label	Genotypic label	Association with colorectal cancer
Streptococcus bovis biotype I	Streptococcus gallolyticus	Strong
Streptococcus bovis biotype II.1	*Streptococcus infantarius* and *Streptococcus lutetiensis*	Weak
Streptococcus bovis biotype II.2	Streptococcus pasteurianus	Weak

As only SGG seems to possess virulence factors that are associated with underlying colonic malignancies, this association suggests a potential risk stratification of patients with SBSEC bacteremia. By accurately identifying those who are truly at risk, meaning only SGG-infected patients, healthcare providers could reduce costs and improve patient safety [20].

However, missing a colon cancer diagnosis can have grave consequences and the overall risk of colonic malignancy in patients with SBSEC infection, particularly endocarditis, requires consideration. The risk of colonoscopy complications is low and is mainly elevated in polypectomy; therefore, a diagnostic evaluation in these patients is a reasonable choice.

Our case report has several limitations that warrant consideration. First, a colonoscopy was not performed earlier, even though the patient presented with anemia. Age-appropriate screening was not done either. These could have potentially facilitated an earlier diagnosis. Second, endoscopic images and computed tomography (CT) scans of the abdomen, which are part of current standard pre- and post-resection assessment practices, are not available because the patient's hospitalization occurred five years ago, and the visual records have since been lost. Detailed records of the cardiothoracic surgery are also unavailable, as the procedure was conducted at a separate institution, and the relevant documents have been misplaced. In addition, the surgical intervention involved an open colectomy rather than a laparoscopic-assisted approach, which is less consistent with contemporary surgical standards that emphasize minimally invasive techniques. Finally, the patient was lost to follow-up and did not show up on the planned third-month visit.

## Conclusions

The case we report highlights the importance of a multidisciplinary approach and sustained clinical vigilance in managing complex infections. While SGP endocarditis associated with colonic malignancy is less expected compared to other subspecies and the association is notably stronger with SGG-endocarditis, a possible heightened risk of colon cancer in all patients with SBSEC-related bacteremia is suggested although this risk is not yet fully quantified. In light of this, considering additional findings, such as endocarditis and anemia, might be helpful in making the decision for a colonoscopy. Although the benefits of screening for colon cancer in the context of endocarditis must be weighed against the risks, early detection of colon cancer could have significant implications for patient outcomes. Our case reinforces the consideration for colonoscopy in patients presenting with SBSEC endocarditis, taking into account the individual clinical scenario.
